# Anti-inflammatory effects of *Lactobacillus johnsonii* L531 in a pig model of *Salmonella* Infantis infection involves modulation of CCR6^+^ T cell responses and ER stress

**DOI:** 10.1186/s13567-020-00754-4

**Published:** 2020-02-24

**Authors:** Gui-Yan Yang, Bing Xia, Jin-Hui Su, Ting He, Xiao Liu, Liang Guo, Shuai Zhang, Yao-Hong Zhu, Jiu-Feng Wang

**Affiliations:** College of Veterinary Medicine, China Agricultural University, Beijing, 100193 China

## Abstract

Probiotic pretreatment is an effective non-antibiotic strategy for preventing or controlling *Salmonella* infections. We found that *Lactobacillus johnsonii* L531, isolated from the colon of a clinically healthy weaned piglet, effectively prevented infection with *Salmonella enterica* serovar Infantis in a pig model. Newly weaned piglets were intragastrically administered *Lactobacillus johnsonii* L531 at 1.0 × 10^10^ CFU/day for 1 week before *S*. Infantis challenge. Pretreatment with *L. johnsonii* L531 lessened the severity of diarrhea and ileal inflammation in *S*. Infantis–infected piglets. Lactobacilli were more abundant in the ileum than jejunum after *L. johnsonii* L531 pretreatment. Treatment with *L. johnsonii* L531 reduced the abundance of total bacteria in the ileal mucosa and the production of lipocalin 2 in the jejunum of piglets challenged with *Salmonella*. Both intestinal morphology and transmission electron microscopy results indicated that *L. johnsonii* L531 alleviated intestinal tissue damage following *S*. Infantis challenge, especially in the villus and endoplasmic reticulum (ER). ER stress induced by *S*. Infantis was attenuated by *L. johnsonii* L531 treatment. The number of CD4^−^ CCR6^+^ T cells decreased following *S.* Infantis challenge, but the percentage of CCR6^−^ IFNγ^+^ T cells in peripheral blood increased. In intestinal mesenteric lymph nodes, *S*. Infantis increased the proportion of CCR6^+^ IFNγ^+^ T cells, whereas *L. johnsonii* L531 induced an increase in the proportion of CD4^+^ CCR6^+^ T cells in response to *S*. Infantis infection. Our data thus suggest that *L. johnsonii* L531 contributes to the maintenance of intestinal homeostasis by modulating T-cell responses and ER stress.

## Introduction

*Salmonella enterica* serovar Infantis, a zoonotic, non-typhoidal *S. enterica* serovar, is a significant cause of diarrhea and gastroenteritis in humans [1]. Several reports have also identified *S*. Infantis in pigs with diarrhea [2, 3]. A recent study reported a prevalence of *S*. Infantis in pigs with *Salmonella*-associated diarrhea in China of 3.85% [3]. The clinical presentation of *S.* Infantis infection, particularly diarrhea, is associated with dysbiosis [4]. Gut-microbiota dysbiosis is more likely to induce intestinal inflammation, which could in turn further exacerbate microbiota dysbiosis.

Unresolved endoplasmic reticulum (ER) stress in the intestine can be both a primary cause and consequence of mucosal inflammation. Intestinal barrier dysfunction due to ER stress induces an inflammatory response involving both innate and adaptive immunity, including activation of Th1 and Th17 responses [5]. The expression of mucosal Th17 signature genes (e.g., CC-chemokine ligand [CCR6]) is increased in *Winnie* mice with severe ER stress and colitis [5]. We found that *S*. Infantis induces an increase in the proportion of proinflammatory CD4^+^ IFNγ^+^ T cells in Peyer’s patches in pigs; however, little is known regarding the roles of CD4^+/−^ CCR6^+/−^ T cells or CCR6^+/−^ IFNγ^+/−^ T cells during *Salmonella* infection.

A proper ER stress response, known as the unfolded protein response (UPR), is important for maintaining homeostasis in the intestinal epithelium, which contains cells that exhibit high protein turnover, such as secretory goblet cells and Paneth cells [6]. Resolution of ER stress by the UPR involves activation of the IRE1, PERK, and ATF6 pathways [6]. It is now clear that microbes themselves and/or their products can directly affect ER stress pathways, particularly IRE1 [6]. Bacteria such as Shiga toxigenic *Escherichia coli* secrete AB_5_ cytotoxins that activate ER stress pathways via direct cleavage of glucose-regulated protein 78 (GRP78) [7]. GRP78, also known as binding immunoglobulin protein (BiP), functions both as a chaperone for misfolded proteins in the ER lumen and as a negative regulator of IRE1 [6]. GRP78 binds to ER receptors (e.g., IRE1), blocking their activation [8]. However, the link between *Salmonella* infection and ER stress in the intestinal mucosa remains to be elucidated.

Treating non-typhoidal *S*. *enterica* infections with antimicrobial agents poses a serious threat to public health due to the development of multidrug resistance. The potential antimicrobial effects of *L. johnsonii* against major gastric and enteric bacterial pathogens have been well characterized [9]. We recently reported that an *L. johnsonii* L531 isolate exhibiting probiotic properties is effective for treating *Salmonella* infections in piglets [10]. In addition, it was recently shown that *L. acidophilus* attenuates mouse colitis by alleviating ER stress [11]. However, the role of probiotic bacteria in modulating ER stress during *Salmonella* infection has not been clearly established.

In the current study, we investigated the effect of selected *L. johnsonii* L531 on the intestinal ER stress response and development of inflammation in a pig model of *S*. Infantis infection. We hypothesized that pretreatment with *L. johnsonii* L531 increases intestinal resistance against *Salmonella* via inflammation-associated T-cell responses.

## Materials and methods

### Ethics statement

Experiments with pigs were carried out in strict accordance with the Guidelines for Laboratory Animal Use and Care from the Chinese Center for Disease Control and Prevention and the Rules for Medical Laboratory Animals from the Chinese Ministry of Health, under protocol CAU20161016-1, approved by the Animal Ethics Committee of China Agricultural University.

### Animals

Eighteen healthy, *Salmonella*-free, Landrace × Large White piglets of mixed gender, selected from 8 different litters, weaned at 21 days of age, and weighing 5.84 ± 0.07 kg, were purchased from Beijing Hog Raising and Breeding Center. The piglets were newly weaned and transported to the animal experimental facility of the College of Veterinary Medicine, China Agricultural University. Each animal was penned separately and had ad libitum access to antibiotic-free feed and water from day 0 (when newly weaned) and until day 18 (when euthanized).

### Bacteria preparation and growth conditions

*Lactobacillus johnsonii* L531, which was isolated from the colonic contents of healthy piglets, was grown in De Man, Rogosa, and Sharpe (MRS) broth (Oxoid, Hampshire, UK) overnight at 37 °C under microaerophilic conditions, as previously described [10]. Bacteria were pelleted by centrifugation at 3000 ×* g* for 10 min at 4 °C and resuspended in physiologic saline, and the concentration was adjusted to 10^9^ CFU/mL.

*Salmonella* Infantis CAU1508 was isolated from the intestinal contents of weaned pigs with diarrhea. The challenge strain, *S*. Infantis CAU1508 (mCherry), was prepared as previously described [12]. Briefly, *S*. Infantis was grown to mid-log phase in fresh Luria–Bertani broth (Oxoid, Basingstoke, UK) at 37 °C with shaking. The *Salmonella* were then harvested by centrifugation at 3000 × *g* for 10 min at 4 °C and resuspended in physiologic saline. An inoculum of *S*. Infantis CAU1508 (mCherry) containing 1.0 × 10^11^ CFU/mL was prepared and quantified by determination of CFUs after plating serial dilutions of bacterial suspensions onto xylose lysine tergitol 4 (XLT4; Beijing Land Bridge Technology Co., Beijing, China) agar plates.

### Experimental design

On the day of weaning (day 0), the piglets were divided into 3 groups (*n* = 6 per group) according to weight and ancestry: (1) control (CN) group (intragastric administration of sterile physiologic saline only); (2) *S*. Infantis (SI) group (intragastric administration of sterile physiologic saline and *S*. Infantis); and (3) *L. johnsonii* +* S*. Infantis (L.j. + SI) group (intragastric administration of *L. johnsonii* and *S*. Infantis). All animals were inoculated intragastrically with test material without sedation, as previously described [13]. Before the study, piglets were determined to be free of *Salmonella* by analysis of feces and blood, as previously described [10]. In brief, fresh fecal samples were plated on XLT4 agar plates after pre-enrichment in buffered peptone water (Beijing Aoboxing Bio- tech Co., Beijing, China). Serum samples were tested for antibodies against *Salmonella* using a porcine *Salmonella* antibody enzyme-linked immunosorbent assay (ELISA) kit (B, C1, and D combined) (Biocheck, London, UK). The results were negative for all piglets.

At 9:00 A.M. from days 1 through 7, L.j. + SI piglets were administered 10 mL of *L. johnsonii* L531 (10^9^ CFU/mL) once daily, whereas piglets in groups CN and SI were administered 10 mL of sterile physiologic saline. At 9:00 A.M. on day 8, piglets in the SI and L.j. + SI groups were inoculated intragastrically with 10 mL of *S*. Infantis (1.0 × 10^11^ CFU/mL), whereas CN piglets received 10 mL of sterile physiologic saline [10].

The severity of diarrhea following *S*. Infantis challenge was scored as previously described [12, 14]. On days 1, 8, 15, and 18, piglets were weighed. Ten days post-infection (day 18), the piglets were euthanized and tissue samples were immediately collected.

### Lactobacilli enumeration

One gram each of intestinal contents (jejunum, caecum and colon), mucosal tissues and systemic organs (liver, spleen and mesenteric lymph nodes [MLNs]) from each animal were homogenized in 9 mL of sterile saline. Serial dilutions were then plated on MRS agar plates for lactobacilli culture (Beijing Land Bridge Technology Co.) and incubated under anaerobic conditions for 48 h at 37 °C. Results are expressed as log_10_ CFU/g contents or log_10_ CFU/g tissue. All counts were performed in triplicate.

### PCR quantification of 16S rRNA genes

Quantitative PCR was performed as previously described, with minor modifications [13]. The following primer sets were used: “all bacteria”, 5′-CGGTGAATACGTTCCCGG-3′, 5′-TACGGCTACCTTGTTACGACTT-3′; *Lactobacillus*: 5′-AGCAGTAGGGAATCTTCCA-3′, 5′-CACCGCTACACATGGAG-3′; *Clostridia*: 5′-ACTCCTACGGGAGGCAGC-3′, 5′-GCTTCTTTAGTCAGGTACCGTCAT-3′.

### Enzyme-linked immunosorbent assay (ELISA)

The concentrations of lipocalin 2 in jejunal and ileal tissues were measured by porcine-specific commercially available ELISA kits (Xuejiete technology co., Beijing, China). The experimental procedures were based on the manufacturer’s instructions.

### Histologic assessment

Proximal, mid-, and distal segments of the jejunum and ileum (approximately 10 × 15 × 3 mm) were fixed in 4% paraformaldehyde and embedded in paraffin. Intestinal pathology was evaluated on hematoxylin & eosin–stained jejunal and ileal sections by a single blinded scorer using a validated scoring system [12, 15]. Villus length and crypt depth of the jejunum were measured using Image-Pro Plus software (version 6.0; Media Cybernetics, Silver Spring, MD, USA). Ten well-oriented villi were selected for each intestinal section (30 measurements for each sample).

### Transmission electron microscopy

Jejunal and ileal tissue samples were cut into fragments of approximately 1 mm^3^ and fixed in 2.5% glutaraldehyde (pH 7.4) for 24 h at room temperature. The fixed tissues were post-fixed in 1% osmium tetroxide, dehydrated using a graduated ethanol series (30, 50, 70, 80, 90 and 100%), embedded in Epon (Energy Beam Sciences, Agawam, MA, USA), sliced into ultrathin section (50–60 nm) using a Leica EM UC6 ultramicrotome (Leica Microsystems, Wetzlar, Germany) and stained with 3% uranyl acetate and lead citrate. The ultrathin sections were observed under an H7500 transmission electron microscope (Hitachi, Tokyo, Japan).

### Flow cytometry

A 3-mL aliquot of peripheral blood from each piglet was collected in Venoject glass tubes containing EDTA (Terumo Europe NV, Leuven, Belgium) prior to *S*. Infantis challenge (0 h) and at 24 and 192 h post-challenge. Intestinal MLN samples were collected immediately after animals were euthanized. Single-cell suspensions of peripheral blood and MLNs were prepared as previously described [16, 17]. Cell viability was evaluated by trypan blue exclusion. A total of 1 × 10^6^ cells were used for staining in each reaction, and more than 2 × 10^4^ gated events per condition were acquired. The following monoclonal antibodies were used: mouse anti–pig CD3ε (clone BB23-8E6-8C8, PerCP-Cy5.5–conjugated, 561478; BD Biosciences), mouse anti–pig CD4α (clone 74-12-4, FITC-conjugated, 559585; BD Biosciences), mouse anti–human CCR6 (clone G034E3, phycoerythrin [PE]-conjugated, 353410; Biolegend), and mouse anti–pig IFN-γ (clone P2G10, AlexaFluor 647–conjugated, 561480; BD Biosciences). Isotype controls FITC-conjugated mouse IgG2b, κ (559532; BD Biosciences), PE-conjugated mouse IgG2b, κ (400311; Biolegend), AlexaFluor 647–conjugated mouse IgG1, κ (557732; BD Biosciences) and fluorescence-minus-one (FMO) controls were included. The stained cells were analyzed on a FACScalibur™ flow cytometer (BD Biosciences), and data analysis was performed using FlowJo 9.3 software (Tree Star) [12].

### Quantitative real-time PCR

The middle jejunum (without Peyer’s patch involvement) and ileum segments collected from each piglet were flash frozen in liquid nitrogen and then stored at −80 °C. Total RNA was extracted from frozen tissues using an EASYspin plus RNA extraction kit (Aidlab Biotechnologies, Beijing, China) according to the manufacturer’s instructions. Reverse transcription and real-time polymerase chain reaction (PCR) analyses were performed as previously described [13]. The cycle threshold (C_T_) values of the target genes (CHOP, GRP78 and CCL2) were normalized to the geometric mean C_T_ values of three reference genes: β-actin, glyceraldehyde-3-phosphate dehydrogenase (GAPDH) and hypoxanthine phosphoribosyl-transferase [15]. Results are presented as fold change using the 2^−ΔΔCT^ method. Primers are listed in Additional file 1.

### Western blotting

Proteins were extracted from the jejunum and ileum sections using Radio-Immunoprecipitation Assay buffer (Sigma-Aldrich, St. Louis, MO, USA), as previously described [13]. Protein concentrations were determined using the BCA method (Thermo Fisher Scientific, Waltham, MA, USA). Proteins were boiled in loading buffer at 100 °C for 5 min, and an equal amount of 20 μg of protein was then loaded onto a sodium dodecyl sulfate–polyacrylamide gel electrophoresis gel. A 10% acrylamide separating gel (pH 8.8) and a 5% acrylamide stacking gel (pH 6.8) were used. The primary antibodies used were polyclonal rabbit anti–pig GRP78/BiP (ab21685, Abcam), polyclonal rabbit anti–pig IRE1α (phospho S724, ab48187; Abcam) and monoclonal mouse anti-GAPDH (60004-1-Ig, Proteintech). Horseradish peroxidase–conjugated secondary antibodies used were goat anti–rabbit IgG (H + L) (SA00001-2; Proteintech) and goat anti–mouse IgG (H + L) (SA00001-1; Proteintech). Immobilon Western chemiluminescent HRP substrate (Millipore, Billerica, MA, USA) was used for immunoblot detection. Bands were visualized using a Tanon-5200 gel image system (Tanon, Shanghai, China). Band intensity was quantified by densitometric analysis using ImageJ software (National Institutes of Health, Bethesda, MD, USA). Results are presented as the ratio of the intensity of the GRP78 or IRE1α band to that of the GAPDH band.

### Statistical analysis

**Figure 1:**
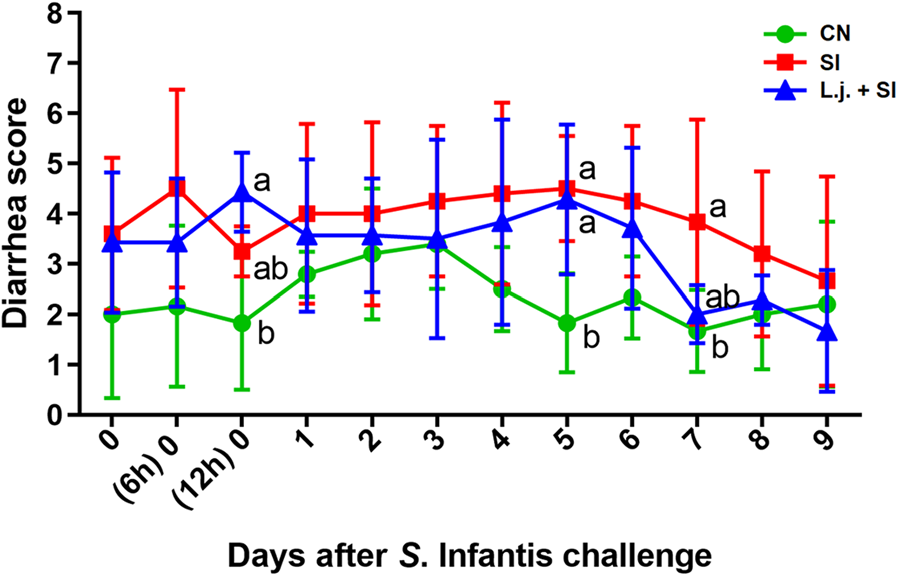
**Effect of*L. johnsonii*L531 pretreatment on the occurrence of*S*. Infantis–induced diarrhea.** Piglets (*n* = 6 per group) received sterile physiologic saline intragastrically (CN), sterile physiologic saline intragastrically followed by *S*. Infantis (1.0 × 10^11^ CFU/mL, 10 mL) challenge (SI), or were pretreated with *L. johnsonii* L531 (1.0 × 10^9^ CFU/mL, 10 mL once daily) for 1 week followed by *S*. Infantis challenge (L.j. + SI). Mean values at the same time point without a common superscript (^a, b^) differ significantly (*P* < 0.05; Tukey’s test). Bars represent mean ± SD.

The SAS statistical software package, version 9.3 (SAS Institute Inc., Cary, NC, USA), and the software’s PROC MIXED procedure were used for statistical analyses. For non-normally distributed and repeated-measure data analysis, the non-parametric Friedman’s test using the SAS procedure FREQ was performed to compare diarrhea scores between treatments. Moreover, the non-parametric Wilcoxon–Mann–Whitney *U*-test was performed to compare differences between treatments in histologic scores for the small intestine. The statistical significance of differences between groups was determined by two-tailed Student’s *t* test or one-way analysis of variance. Data are presented as the mean ± standard error of the mean (SEM), except for Figure 1. *P*-values: *P < 0.05; **P < 0.01; ***P < 0.001.

## Results

### Effect of *L. johnsonii* L531 pretreatment on diarrhea incidence in piglets infected with *S*. Infantis

Administration of *L. johnsonii* L531 reduced the incidence of postweaning diarrhea compared with untreated CN piglets (*P* = 0.014; Additional file 2). Following *S*. Infantis challenge, all piglets in the SI group exhibited diarrhea lasting for more than 2 days, whereas only 3 piglets pretreated with the probiotic developed diarrhea. On day 7 post-challenge, diarrhea scores were higher in SI piglets than CN piglets (*P* = 0.026). However, at 12 h and 5 days after infection, piglets pretreated with *L. johnsonii* exhibited higher diarrhea scores compared with CN piglets (Figure 1).

### *L. johnsonii* L531 pretreatment decreased the abundance of total bacteria in response to *S*. Infantis challenge

**Figure 2:**
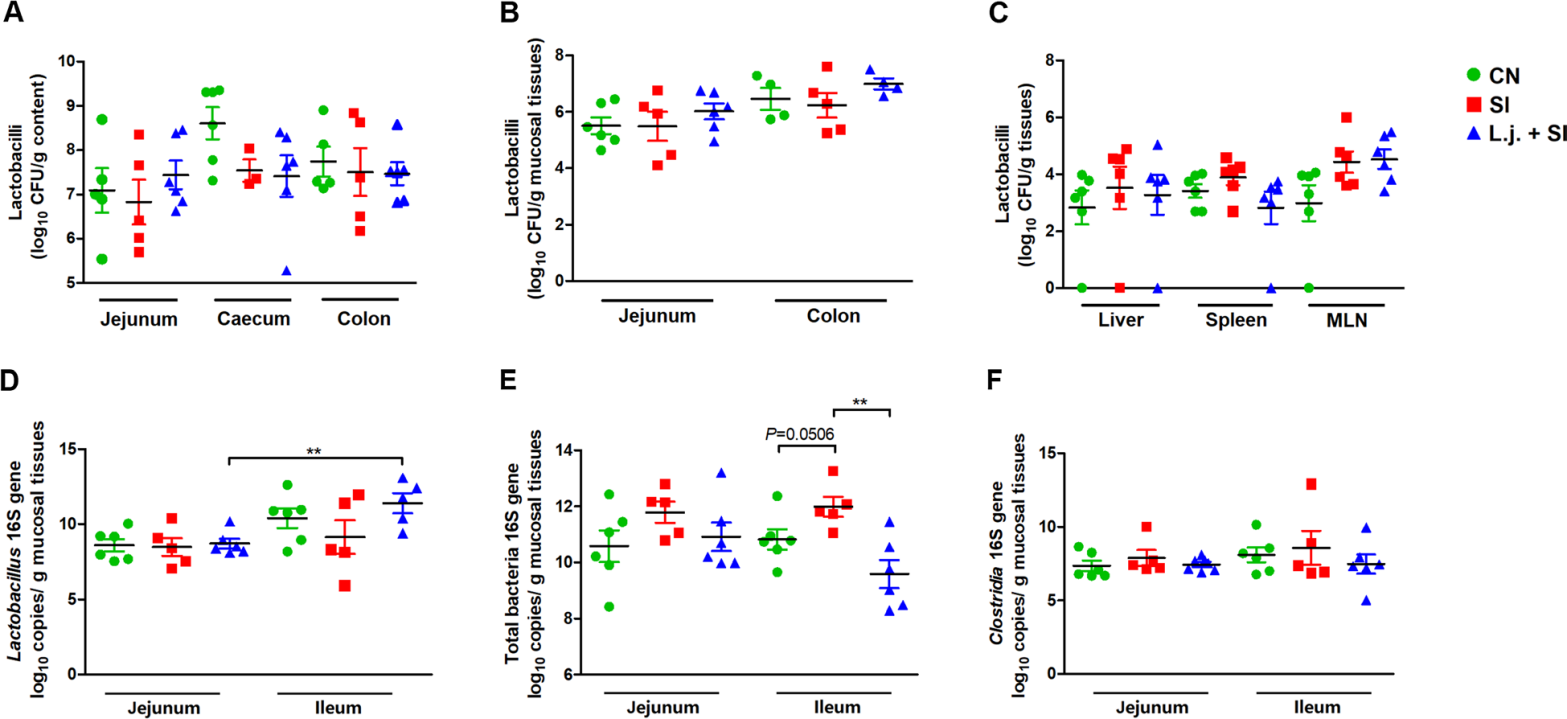
***L. johnsonii*L531 pretreatment decreased the abundance of total bacteria in the ileal mucosa.** Mucosal tissues (midjejunum, distal ileum, caecum, and colon) and systemic organs (liver, spleen, and MLNs) were collected from the indicated piglets 10 days after *S*. Infantis challenge. The number of lactobacilli (**A**–**C**) in intestinal mucosal tissues and systemic organs after *S*. Infantis challenge was monitored using culture-based enumeration. Bacterial DNA isolated from 200 mg of intestinal mucosal tissues from piglets of each of the three groups was analyzed by quantitative PCR using universal primers for bacterial 16S rRNA genes (**D**–**F**). Data are presented as mean ± SEM. *P < 0.05; **P < 0.01.

The number of lactobacilli was similar in different intestinal sections and systemic organs (e.g., liver, spleen, and MLNs) (Figures 2A–C). However, the number of lactobacilli was higher in mucosal tissues of the ileum than the jejunum (*P* = 0.0039; Figure 2D). Interestingly, pretreatment with *L. johnsonii* reduced the *S*. Infantis–induced increase in the abundance of total bacteria in the ileal mucosa (*P* = 0.0039; Figure 2E). No changes in the abundance of *Clostridia* in the small intestine were detected in the present study (Figure 2F).

### *L. johnsonii* L531 pretreatment attenuates the severity of *Salmonella*-induced intestinal damage and inflammation

**Figure 3:**
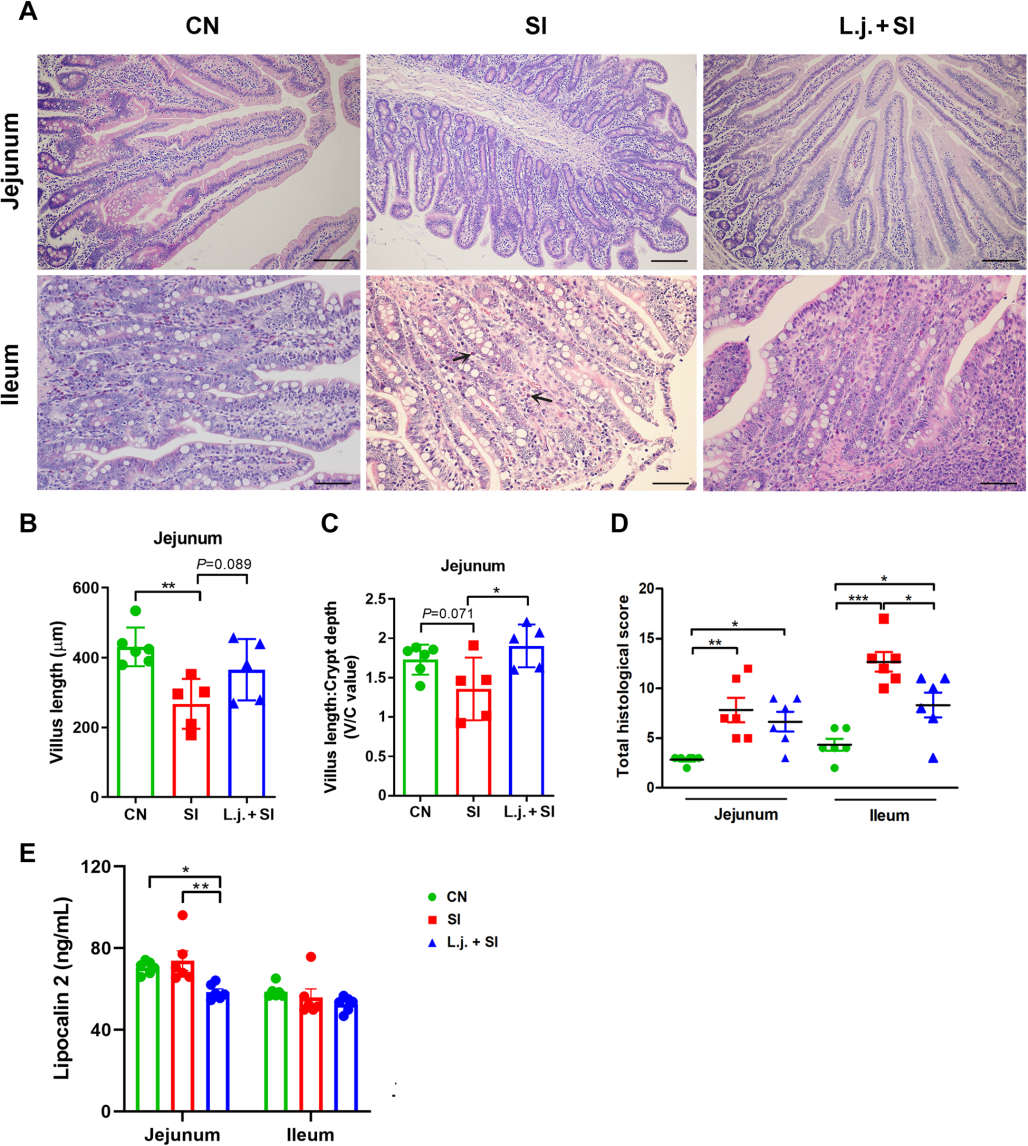
***L. johnsonii*L531 ameliorates the small intestinal inflammation and tissue damage caused by*Salmonella***. **A** Representative photomicrographs of hematoxylin and eosin–stained jejunal and ileal sections. Scale bars, 100 µm. Villus length (**B**) and the ratio of villus length to crypt depth (**C**) at the jejunum of animals of the indicated groups are shown. Arrowheads indicate inflammatory infiltration in the ileum of *S*. Infantis–infected piglets. **D** Jejunal and ileal histologic scores (non-parametric Wilcoxon–Mann–Whitney *U*-test). **E** Level of lipocalin 2 in mucosal tissues of the jejunum and ileum as determined by ELISA. Data are presented as the mean ± SEM for each tissue. *P < 0.05; **P < 0.01; ***P < 0.001 (Tukey’s test).

Analyses of histopathologic changes revealed that infection with *S*. Infantis caused villus loss compared with the control (*P* = 0.002; Figures 3A, B), whereas no differences in villus length were observed in piglets pretreated with *L. johnsonii*. In the jejunum, the villus length/crypt depth ratio was also higher in piglets pretreated with *L. johnsonii* than in SI piglets without probiotic pretreatment (*P* = 0.03; Figure 3C). Histologic assessment of the jejunum and ileum revealed that intragastric administration of *L. johnsonii* tempered the severity of *Salmonella*-associated inflammation in the ileum (*P* < 0.05; Figures 3A, D).

The production of lipocalin 2 in the jejunal mucosa decreased after preatment with *L. johnsonii* compared with CN and SI piglets (*P* < 0.05; Figure 3E).

### Effect of *L. johnsonii* L531 on ER stress induced by *S*. Infantis infection

**Figure 4:**
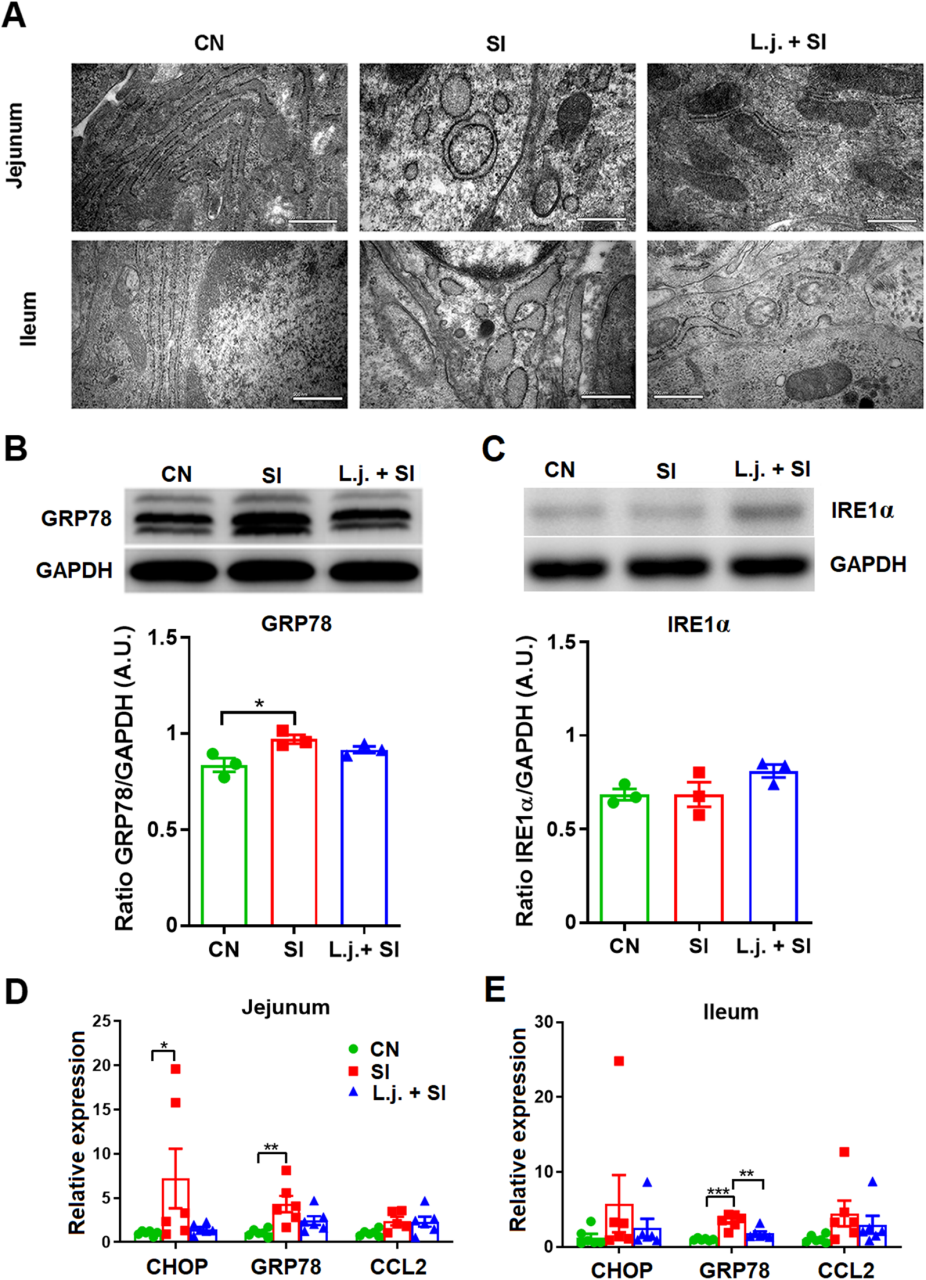
***Lactobacillus johnsonii*L531 pretreatment attenuates*S*. Infantis–induced ER stress in the small intestine. A** Effect of *L. johnsonii* L531 on the structure of the rough ER in the small intestine after *S*. Infantis infection as observed using TEM. **B**, **C** Representative Western blot results for GRP78 and IRE1α in the ileum collected from piglets 10 days after *S*. Infantis challenge (upper panel). Results are presented as the ratio of the GRP78 or IRE1α band intensity to the intensity of the GAPDH band (lower panel). **D**, **E** Expression of ER marker gene mRNAs in both jejunal and ileal tissues collected from piglets 10 days after *S*. Infantis challenge analyzed using quantitative real-time PCR. Data are presented as the mean ± SEM for each tissue. *P < 0.05; **P < 0.01; ***P < 0.001 (Tukey’s test).

The structure and organization of organelles (particularly the ER) in the jejunum and the ileum are shown in Figure 4A. Intestinal epithelial cells in CN piglets exhibited a regular ultrastructure and extensive rough ER surrounding organelles such as mitochondria. In *S*. Infantis–infected piglets, the ER lumen was significantly dilated and the ER structure was damaged. However, *L. johnsonii* pretreatment ameliorated this ultrastructural damage and reduced the dilation of ER lumen.

Expression of GRP78 protein in the ileum was elevated after *Salmonella* challenge compared with the CN group (*P *= 0.033; Figure 4B). However, no changes in the expression of IRE1α were observed in the ileum. There were no differences between groups in the expression of GRP78 and IRE1α in the jejunum (data not shown).

The expression of GRP78 mRNA in the ileum was also upregulated compared with the CN group (*P* < 0.001), and this upregulation was attenuated by *L. johnsonii* pretreatment (*P *= 0.002; Figure 4E). The expression of mRNAs encoding CHOP and GRP78 was higher in the jejunum of *S*. Infantis–infected animals than control piglets (*P* < 0.05; Figure 4D).

### *L. johnsonii* L531 pretreatment increased the number of CD4^+^ CCR6^+^ T cells in MLNs in response to *Salmonella* challenge

**Figure 5:**
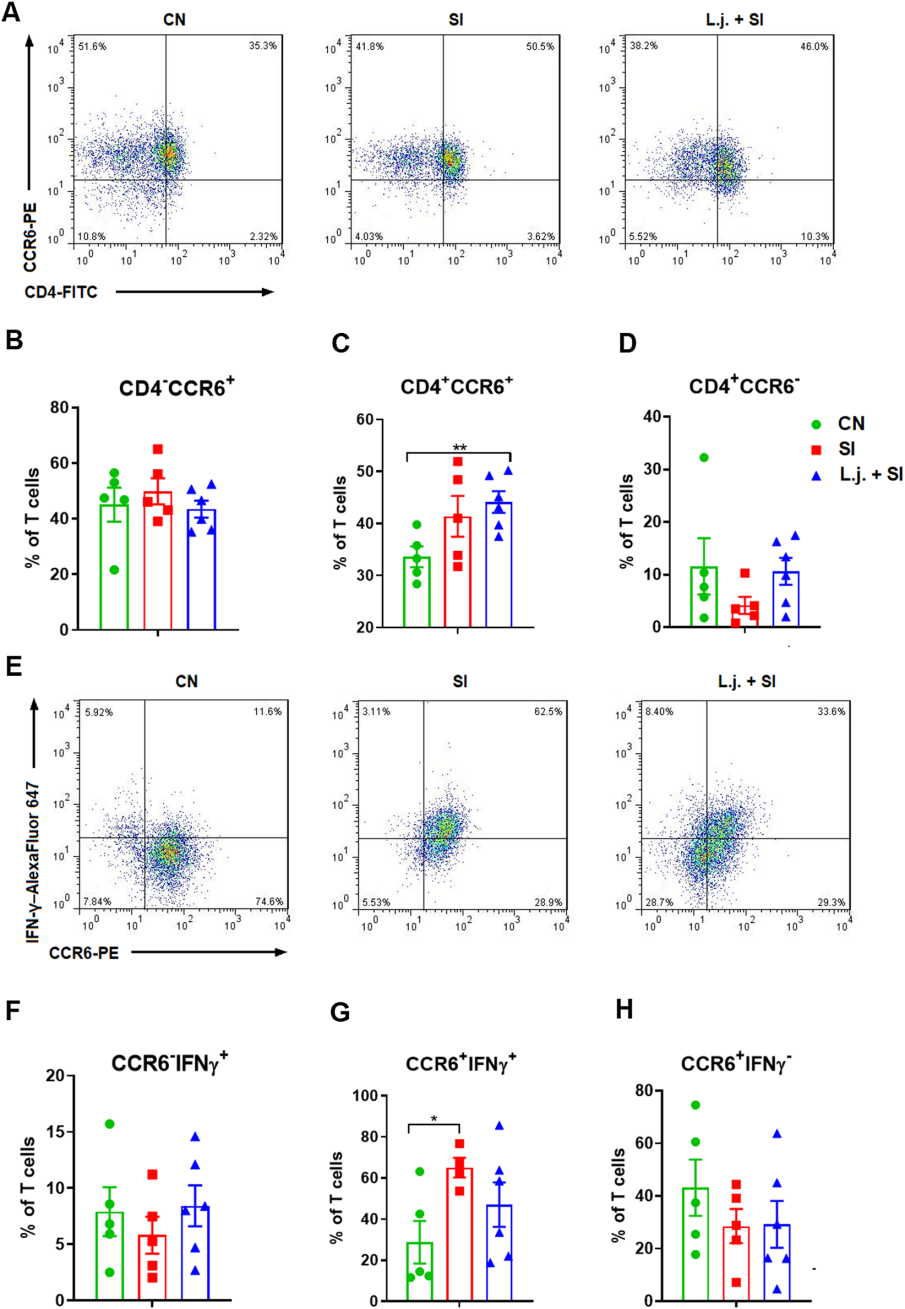
**Effect of*L. johnsonii*L531 pretreatment on CD4**^**+/−**^**CCR6**^**+/−**^**T cells and CCR6**^**+/−**^**IFNγ**^**+/−**^**T cells in intestinal MLNs.** Lymphocytes were isolated from the intestinal MLNs of piglets 10 days after *S*. Infantis challenge. Representative dot plots show the percentages of **A** CD4^+/−^ CCR6^+/−^ cells and **E** CCR6^+/−^ IFNγ^+/−^ cells among CD3^+^ T cells. Flow cytometry analysis of the percentages of **B** CD4^**−**^ CCR6^+^, **C** CD4^+^ CCR6^+^, **D** CD4^+^ CCR6^**−**^ cells and **F** CCR6^−^ IFNγ^+^, **G** CCR6^+^ IFNγ^+^, and **H** CCR6^+^ IFNγ^−^ cells within the CD3^+^ T-cell population in the indicated piglets. Data are presented as the mean ± SEM for each time point. *P < 0.05 (Tukey’s test).

We also assessed changes in the percentages of CD4^+/−^ CCR6^+/−^ T cells and CCR6^+/−^ IFNγ^+/−^ T cells in the MLNs. An increase in the percentage of CD4^+^ CCR6^+^ T cells was observed in response to *Salmonella* challenge in the intestinal MLNs of piglets pretreated with *L. johnsonii* (*P *= 0.006; Figure 5C). In comparison with controls, an increased percentage of CCR6^+^ IFNγ^+^ T cells was observed in SI piglets but not *L. johnsonii*–treated piglets (*P *= 0.023; Figure 5G). Nevertheless, no changes in the percentages of CD4^−^ CCR6^+^ T cells or CCR6^−^ IFNγ^+^ T cells were observed in the MLNs (Figures 5B, F). The detailed gating strategy for assessing lymphocytes from MLNs is shown in Additional file 3.

### *Salmonella* Infantis infection decreased the percentage of CD4^−^ CCR6^+^ T cells but increased the percentage of CCR6^−^ IFNγ^+^ T cells

**Figure 6:**
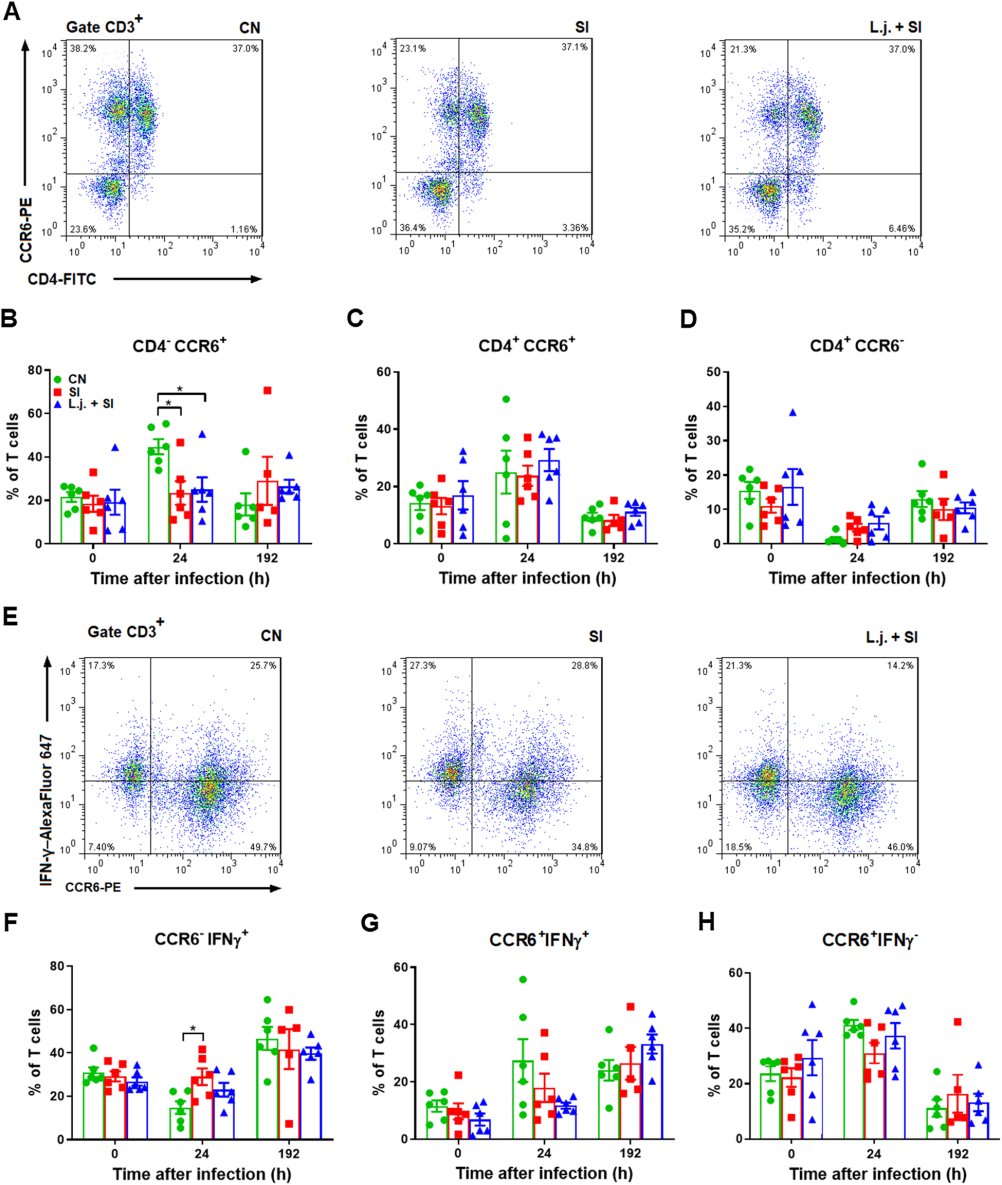
**Effect of*L. johnsonii*L531 pretreatment on CD4**^**+/−**^**CCR6**^**+/−**^**T cells and CCR6**^**+/−**^**IFNγ**^**+/−**^**T cells in peripheral blood.** Peripheral blood samples were collected from the indicated piglets at 0, 24, and 192 h after *S*. Infantis challenge. Representative dot plots show the percentages of **A** CD4^+/−^ CCR6^+/−^ cells and **E** CCR6^+/−^ IFNγ^+/−^ cells among CD3^+^ T cells at 24 h after infection. Flow cytometry analysis of the percentages of **B** CD4^**−**^ CCR6^+^, **C** CD4^+^ CCR6^+^, **D** CD4^+^ CCR6^**−**^ cells and **F** CCR6^−^ IFNγ^+^, **G** CCR6^+^ IFNγ^+^, and **H** CCR6^+^ IFNγ^−^ cells within the peripheral CD3^+^ T-cell population in the indicated piglets. Data are presented as the mean ± SEM for each time point. *P < 0.05 (Tukey’s test).

To examine the role of CCR6^+^ T cells in reponse to *Salmonella* infection, we investigated the percentage of CD4^+/−^ CCR6^+/−^ T cells in peripheral blood at 0, 24, and 192 h following *S*. Infantis challenge. At 24 h after *S*. Infantis infection, the percentage of peripheral blood CD4^−^ CCR6^+^ T cells was decreased compared with the control (*P* < 0.05; Figure 6B). However, the percentage of CD4^+^ CCR6^+^ T cells and CD4^+^ CCR6^−^ T cells did not change significantly, even in piglets pretreated with probiotic (Figures 6C, D).

To further define the CCR6^+/−^ T-cell response to *Salmonella*, T cells were analyzed by flow cytometry for the production of IFNγ. The percentage of CCR6^−^ IFNγ^+^ T cells was higher in the peripheral blood of SI pigs compared with CN pigs at 24 h after *Salmonella* challenge (*P *= 0.032; Figure 6F). The percentage of CCR6^+^ T cells producing IFNγ did not significantly change in this study (Figure 6G). The detailed gating strategy for assessing peripheral blood lymphocytes is shown in Additional file 4.

## Discussion

*Lactobacillus johnsonii* L531, isolated from the intestinal contents of healthy piglets, effectively reduced the incidence of diarrhea after *Salmonella* infection in pretreated piglets. Pretreatment with *L. johnsonii* prevented *S*. Infantis–induced weight loss and reduced the degree of intestinal colonization by *Salmonella* [10]. Consistent with this result, *L. johnsonii* L531 pretreatment attenuated the *S*. Infantis–induced increase in the abundance of total bacteria in the ileum, suggesting that *L. johnsonii* L531 could be useful in the management of bacterial overgrowth in the small intestine [18].

The results of the present study suggest that *Lactobacillus* cells are more abundant in the ileum than the jejunum. Consistent with this observation, histologic assessment of the small intestine demonstrated that *L. johnsonii* L531 pretreatment significantly reduced the severity of *Salmonella*-associated inflammation in the ileum. In addition, *L. johnsonii* L531 pretreatment also protected animals from *S*. Infantis–induced intestinal damage (villus loss and ultrastructural damage). This was consistent with a previous report that probiotic supplementation increases the villus height and crypt depth in the intestine of piglets [19].

The current study investigated the effect of *L. johnsonii* L531 in regulating the expression of ER stress markers (GRP78, CHOP, and p-IRE1) in the small intestine of piglets with *Salmonella* infection, based on a previous study [20]. *Salmonella* Infantis upregulates the intestinal mRNA expression of CHOP, a factor that promotes programmed cell death, and elevates the expression of GRP78 in the small intestine, which is indicative of ER stress. Overexpression of GRP78 attenuates activation of IRE1 [21]. The IRE1 branch is thought to be the last arm of the UPR to be activated, with PERK being the first, closely followed by ATF6 [22]. The UPR activates lipocalin 2 production in prostate cancer cells [23]. Lipocalin 2 was also found to induce apoptosis under ER stress in lung cancer cells [24]. Of note, *L. johnsonii* L531 suppressed the intestinal expression of GRP78 and inhibited the production of inflammatory lipocalin 2 in the jejunum of piglets infected with *Salmonella*. It has been consistently demonstrated that *L. acidophilus* attenuates mouse colitis by inhibiting ER stress [11]. Taken together, these results indicate that *L. johnsonii* L531 suppresses ER stress, which contributes to the amelioration of intestinal inflammation caused by *Salmonella* involving reducing lipocalin 2 production.

Lipocalin 2 also plays a role in inflammation development by enhancing the Th 17 response [25]. A previous study demonstrated that the Th1/Th17 cell population is enriched in response to exposure to *E. coli* and *L. rhamnosus* [26]. In mice, several CD4^+^ CCR6^+^ T-cell subpopulations can typically be distinguished, including Th17 cells and regulatory T cells [27]. CCR6^−/−^ mice exhibit a significantly reduced frequency of Th17 cells and expression of Th17-related cytokines in the Peyer’s patches [28]. CCR6 deficiency reduces IL-22 expression and subsequently decreases the production of antimicrobial peptides [28]. However, less-severe intestinal pathology was observed in DSS-treated mice with CCR6 deficiency [29]. In our study, *S*. Infantis–infected piglets exhibited a significantly lower percentage of CD4^−^ CCR6^+^ T cells in the circulation compared with control piglets, indicating chemoattraction of CD4^−^ CCR6^+^ T cells from the circulation toward the peripheral tissues. *Salmonella* Infantis increases the populations of CCR6^+^ IFNγ^+^ T cells (e.g., Th1/Th17 cells) in the MLNs and CCR6^−^ IFNγ^+^ T cells (e.g., Th1 cells) in the peripheral blood. Pretreatment with *L. johnsonii* L531 induced an increase in the proportion of CD4^+^ CCR6^+^ T cells (e.g., Th17 cells and regulatory T cells) in MLNs in response to *S*. Infantis challenge. We previously found that excessive Th1 immune responses in the peripheral blood contribute to systemic inflammation caused by *S*. Infantis [12]. Our data indicate that the increase in the percentage of CD4^+^ CCR6^+^ T cells promoted by *L. johnsonii* L531 provides protection from intestinal inflammation during *S*. Infantis infection. However, the precise roles played by CD4^+/−^ CCR6^+^ T cells and their cytokines in response to *Salmonella* infection remain to be elucidated.

**Figure 7:**
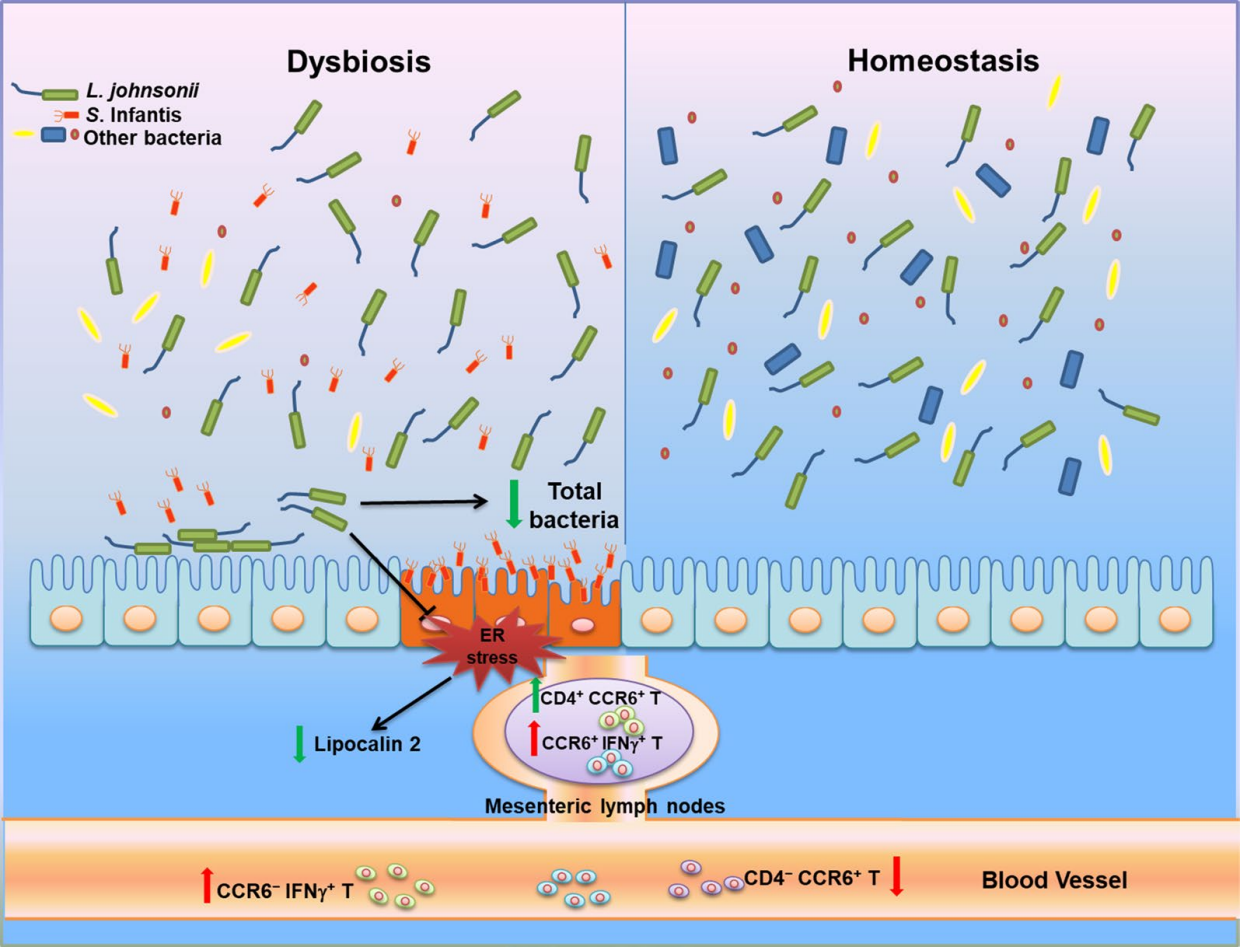
**Pretreatment with probiotic*L. johnsonii*L531 protects the intestine from*S*. Infantis infection by regulating ER protein expression and CCR6**^**+**^**T-cell responses.***Salmonella* Infantis infection is usually associated with dysbiosis [4]. Intragastric administration of *L. johnsonii* L531 decreased the abundance of total bacteria and reduced the colonization of *Salmonella* in the intestine [10]. During *S*. Infantis infection, *L. johnsonii* L531 attenuates ER stress and inhibits the production of lipocalin 2 in the intestine. In the intestinal mesenteric lymph nodes, *S*. Infantis elevated the proportion of CCR6^+^ IFNγ^+^ T cells, and *L. johnsonii* L531 induced an increase in CD4^+^ CCR6^+^ T cells in response to *S*. Infantis challenge. Challenge with *S*. Infantis decreased the percentage of CD4^−^ CCR6^+^ T cells but increased the percentage of CCR6^−^ IFNγ^+^ T cells in peripheral blood. The *Salmonella*-associated ER stress ameliorated by *L. johnsonii* L531 and its regulation of T cell responses during *S*. Infantis infection contribute to inhibition of inflammation development and maintenance of intestinal homeostasis.

In conclusion, pretreating piglets with *L. johnsonii* L531 reduces the severity of *Salmonella*-induced diarrhea. In addition, *L. johnsonii* L531 pretreatment attenuates *Salmonella*-induced outgrowth of total bacteria in the intestine. In ameliorating intestinal inflammation and preventing *Salmonella*-induced tissue damage, *L. johnsonii* L531 inhibits ER stress and the production of lipocalin 2 in the intestinal mucosa in concert with CCR6^+^ T-cell responses (Figure 7).

## Supplementary information

**Additional file 1. Sequences of oligonucleotide primers used for quantitative real-time PCR, length of the respective PCR product, and gene accession numbers.** The table shows information of oligonucleotide primers used for quantitative real-time PCR in this study.

**Additional file 2. Effects of intragastric administration of*Lactobacillus johnsonii*on the incidence of diarrhea in newly weaned pigs before and after*S*. Infantis challenge.** The table shows the incidence of diarrhea in newly weaned pigs in week 1 before *S*. Infantis challenge and ten days post infection*. L. johnsonii* L531 reduced the incidence of postweaning diarrhea compared with untreated CN piglets (*P* < 0.05). Data are presented as the mean ± SEM (*n* = 6 pigs per group). *P < 0.05; **P < 0.01 (Pearson’s Chi square test).

**Additional file 3. Representative dot plots show the gating strategy for mesenteric lymph nodes lymphocytes.** (A) FSC/SSC dot plot of mesenteric lymph nodes lymphocytes, cells with no gating. (B) CD3 dot plot, cells were gated on lymphocytes. (C) CD4/CCR6 dot plot, cells were gated on CD3^+^. (D) CCR6/IFNγ dot plot, cells were gated on CD3^+^. (E) CD4 dot plot, cells were gated on CD3^+^. (F) IFNγ dot plot, cells were gated on CD3^+^. (G) FMO control (Cells were stained with CD3 and CCR6 but no IFNγ antibodies).

**Additional file 4. Representative dot plots show the gating strategy for peripheral blood lymphocytes.** (A) FSC/SSC dot plot of peripheral blood lymphocytes, cells with no gating. (B) CD3 dot plot, cells were gated on lymphocytes. (C) CD4/CCR6 dot plot, cells were gated on CD3^+^. (D) CCR6/IFNγ dot plot, cells were gated on CD3^+^. (E) FMO control (Cells were stained with CD3 and CCR6 but no IFNγ antibodies).

## Abbreviations

*S*. Infantis: *Salmonella enterica* serovar Infantis
*L. johnsonii*: *Lactobacillus johnsonii*
IL: Interleukin
MLN: Mesenteric lymph nodes
CCL: CC-chemokine ligand
CCR: CC-chemokine receptor
ER: Endoplasmic reticulum
UPR: Unfolded protein response
GRP78: Glucose regulated protein 78
BiP: Binding immunoglobulin protein
