# Supplementary material for: Anti-inflammatory effects of Lactobacillus johnsonii L531 in a pig model of Salmonella Infantis infection involves modulation of CCR6+ T cell responses and ER stress
Source: Vet Res. 2020 Feb 24;51:26. doi: 10.1186/s13567-020-00754-4 (PMC7041187; doi:10.1186/s13567-020-00754-4)
Supplement: Supplementary file 1 — Additional file 1. Sequences of oligonucleotide primers used for quantitative real-time PCR, length of the respective PCR product, and gene accession numbers. The table shows information of oligonucleotide primers used for quantitative real-time PCR in this study. [file 13567_2020_754_MOESM1_ESM.doc]

**Additional file 1 Sequences of oligonucleotide primers used for quantitative real-time PCR, length of the respective PCR product, and gene accession numbers.**

| **Gene**  **product*^a^*** | **Primer** | | **Product**  **size (bp)** | **Accession number** |
| --- | --- | --- | --- | --- |
|  | **Direction*****^b^*** | **Sequence (5'→3')** |  |  |
| HPRT | F | GTGATAGATCCATTCCTATGACTGTAGA | 104 | U69731 |
|  | R | TGAGAGATCATCTCCACCAATTACTT |  |  |
| GAPDH | F | CCAGAACATCATCCCTGCTT | 229 | NM_001206359.1 |
|  | R | GTCCTCAGTGTAGCCCAGGA |  |  |
| β-actin | F | CTCTTCCAGCCCTCCTTCCT | 103 | XM_003357928.2 |
|  | R | GCGTAGAGGTCCTCCTGATGT |  |  |
| CCL2 | F | AGAACCCAAGCAGAAGTGGG | 73 | NM_214214.1 |
|  | R | TCAAGGCTTCGGAGTTTGGTT |  |  |
| Bip | F | CTATGAAGGTGAGCGACCCC | 100 | NM_005347.4 |
|  | R | TTCAATCTGCGGGACTCCAC |  |  |
| CHOP | F | GCTCTGATTGACCGGATGGT | 95 | NM_001144845.1 |
|  | R | GCCACTTCCAGGAAAGGTCA |  |  |

*^a^* HPRT, hypoxanthine phosphoribosyl-transferase; GAPDH, glyceraldehyde-3-phosphate dehydrogenase; CCL, CC-chemokine ligand.

*^b^* F, forward; R, reverse.
