# Supplementary material for: Anti-inflammatory effects of Lactobacillus johnsonii L531 in a pig model of Salmonella Infantis infection involves modulation of CCR6+ T cell responses and ER stress
Source: Vet Res. 2020 Feb 24;51:26. doi: 10.1186/s13567-020-00754-4 (PMC7041187; doi:10.1186/s13567-020-00754-4)
Supplement: Supplementary file 2 — Additional file 2. Effects of intragastric administration ofLactobacillus johnsoniion the incidence of diarrhea in newly weaned pigs before and afterS. Infantis challenge. The table shows the incidence of diarrhea in newly weaned pigs in week 1 before S. Infantis challenge and ten days post infection. L. johnsonii L531 reduced the incidence of postweaning diarrhea compared with untreated CN piglets (P < 0.05). Data are presented as the mean ± SEM (n = 6 pigs per group). *P < 0.05; **P < 0.01 (Pearson’s Chi square test). [file 13567_2020_754_MOESM2_ESM.doc]

**Additional file 2 Effects of** **intragastric administration of** ***Lactobacillus johnsonii* on the incidence** **of diarrhea in newly weaned pigs before and after** ***S*. Infantis challenge.**

| **Group*^a^*/item** | **Pigs** |  |  |  | |  | **Pig days** | |  |  |  |  |
| --- | --- | --- | --- | --- | --- | --- | --- | --- | --- | --- | --- | --- |
|  | **At risk** | **With diarrhea** | | | **At risk** | | **With diarrhea** | |  | **Significance of difference** | | |
|  | (n) | (n) | (%) |  | | (n) | (n) | (%) | SI L.j.+SI | | | |
| Before infection |  |  |  |  | |  |  |  |  |  |  |  |
| CN | 6 | 2 | 33.3% |  | | 42 | 8 | 19.0% |  |  | * |  |
| SI | 6 | 3 | 50.0% |  | | 42 | 16 | 38.1% |  |  |  |  |
| L.j. + SI | 6 | 1 | 16.7% |  | | 42 | 5 | 11.9% |  |  |  |  |
|  |  |  |  |  | |  |  |  |  |  |  |  |
| Post-infection |  |  |  |  | |  |  |  |  |  |  |  |
| CN | 6 | 3 | 50.0% |  | | 60 | 10 | 16.7% |  | ** | * |  |
| SI | 6 | 6 | 100% |  | | 60 | 32 | 53.3% |  |  |  |  |
| L.j. + SI | 6 | 3 | 50.0% |  | | 60 | 25 | 41.7% |  |  |  |  |

*^a^* Piglets received sterile physiologic saline (CN), received sterile physiologic saline followed by *S*. Infantis (1.0 × 10^11^ CFU/mL, 10 mL) challenge (SI), or were pretreated with *L. johnsonii* L531 (1.0 × 10^9^ CFU/mL, 10 mL once daily) for 1 week followed by *S*. Infantis challenge (L.j.+SI). All piglets were maintained by intragastric administration without sedation.

*n* = 6 pigs per group; Pearson’s chi-square test.
