# Supplementary figures and images for: Anti-inflammatory effects of Lactobacillus johnsonii L531 in a pig model of Salmonella Infantis infection involves modulation of CCR6+ T cell responses and ER stress
Source: Vet Res. 2020 Feb 24;51:26. doi: 10.1186/s13567-020-00754-4 (PMC7041187; doi:10.1186/s13567-020-00754-4)

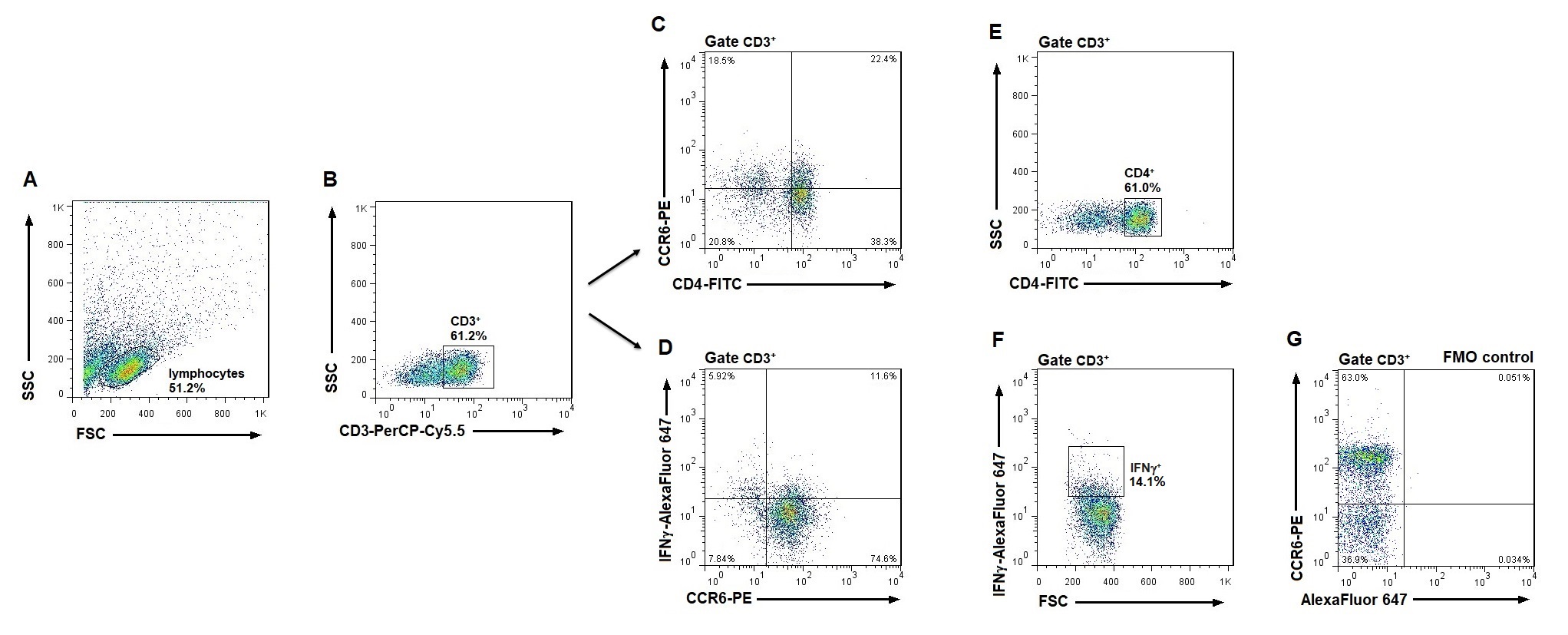

Supplement: Supplementary file 3 — Additional file 3. Representative dot plots show the gating strategy for mesenteric lymph nodes lymphocytes. (A) FSC/SSC dot plot of mesenteric lymph nodes lymphocytes, cells with no gating. (B) CD3 dot plot, cells were gated on lymphocytes. (C) CD4/CCR6 dot plot, cells were gated on CD3+. (D) CCR6/IFNγ dot plot, cells were gated on CD3+. (E) CD4 dot plot, cells were gated on CD3+. (F) IFNγ dot plot, cells were gated on CD3+. (G) FMO control (Cells were stained with CD3 and CCR6 but no IFNγ antibodies). [file 13567_2020_754_MOESM3_ESM.docx]

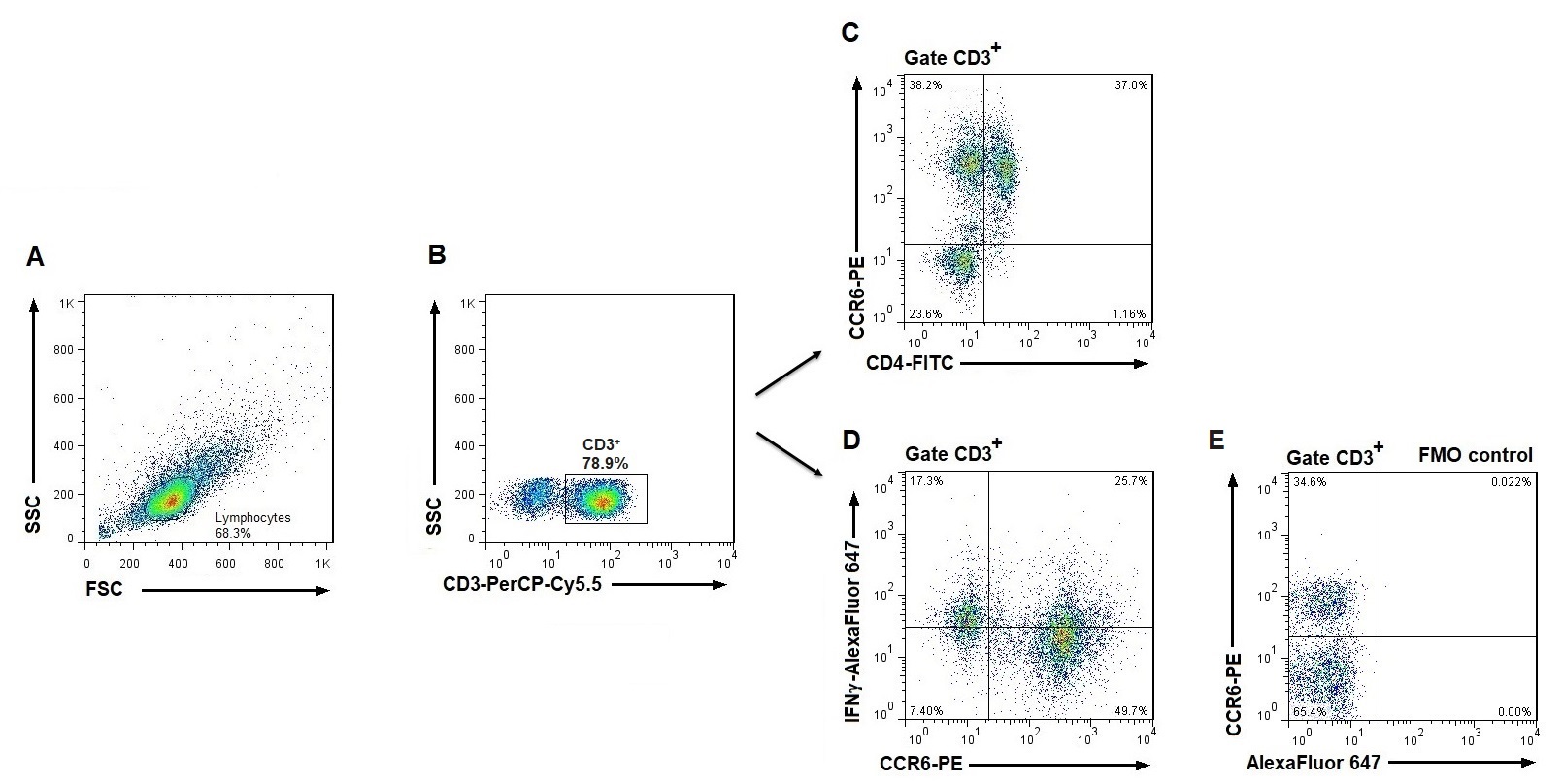

Supplement: Supplementary file 4 — Additional file 4. Representative dot plots show the gating strategy for peripheral blood lymphocytes. (A) FSC/SSC dot plot of peripheral blood lymphocytes, cells with no gating. (B) CD3 dot plot, cells were gated on lymphocytes. (C) CD4/CCR6 dot plot, cells were gated on CD3+. (D) CCR6/IFNγ dot plot, cells were gated on CD3+. (E) FMO control (Cells were stained with CD3 and CCR6 but no IFNγ antibodies). [file 13567_2020_754_MOESM4_ESM.docx]
